# Atmospheric Sensors and Energy Harvesters on Overhead Power Lines

**DOI:** 10.3390/s18010114

**Published:** 2018-01-03

**Authors:** Richard M. White, Duy-Son Nguyen, Zhiwei Wu, Paul K. Wright

**Affiliations:** 1 Department of Electrical Engineering and Computer Sciences, University of California, Berkeley, CA 94720, USA; nguyen.duyson@berkeley.edu; 2 Department of Mechanical Engineering, University of California, Berkeley, CA 94720, USA; zway@berkeley.edu (Z.W.); pwright@me.berkeley.edu (P.K.W.)

**Keywords:** high-efficiency energy harvesters, overhead power lines, flux guides

## Abstract

We demonstrate the feasibility of using novel, small energy harvesters to power atmospheric sensors and radios simply attached to a single conductor of existing overhead power distribution lines. We demonstrate the ability to harvest the required power for operating multiple atmospheric and power-system sensors, together with short-range radios that could broadcast atmospheric sensor data to the cellphones of people nearby. Occasional long-range broadcasts of the data could also be made of both atmospheric and power-line conditions.

## 1. Introduction

The growing recognition of the hazards of atmospheric pollution, and the participation with atmospheric scientists in the development of a particulate matter monitor [1], led us to consider ways of increasing the spatial density of atmospheric measuring points and facilitating the powering of instruments for measuring atmospheric properties. Ideally, one seeks widely distributed, small, and inexpensive communicating instruments powered by continuous power sources. One such solution is operating inexpensive wirelessly-enabled monitors and sensors supported on individual conductors of ubiquitous overhead distribution power-lines.

In what follows we describe a surprisingly efficient energy harvester that couples to the magnetic field that surrounds a conductor on such a line, and we cite measurements that show that the harvester can deliver enough power to simultaneously operate many different atmospheric sensors and monitors, as well as radios to transmit measured data.

## 2. Energy Harvester

In previous research we developed an energy harvester [2] containing a cantilever beam, resonant at 60 Hz, to which permanent magnets were attached and onto which a piezoelelectric lead-zirconate-titanate (PZT) film had been deposited. When this harvester was placed near an alternating current (AC) current-carrying conductor, the cantilever beam vibrated because of coupling of the magnetic field produced by the conductor current to the harvester’s permanent magnets, causing the piezoelectric film to produce an output. Unfortunately, the power produced with this harvester was in the low milliwatt range, and additionally it was known that the piezoelectric property of the PZT material tends to decay with use. Therefore, we have instead investigated a coil-based energy harvester. The coil-based energy harvester has been reported in literature, e.g., [3,4]. However, here we will present the use of high-permeability electrical steel as a flux guide to dramatically increase the output power.

**Figure 1:**
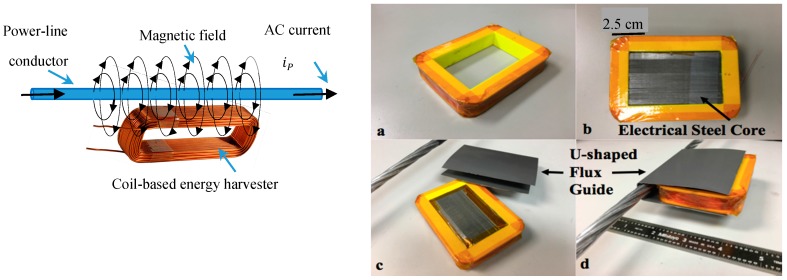
Left: Concept of coil-based energy harvester; magnetic field produce by current flow in a power-line conductor. Note that much of the generated magnetic field does not pass through the coil. Right: (**a**): coil form; (**b**): coil with electrical steel core; (**c**): coil, electrical steel U-shaped flux guide, and a length of stranded aluminum power-line conductor; (**d**): elements from (**c**) assembled (with ruler).

The coil-based harvester is a credit-card sized (8.9 × 6.7 × 1.6 cm) rectangular coil having typically 1250 turns of insulated copper wire wound on a polymeric form (Figure 1a) and provided with a core of “electrical steel”—sheets of high-permeability steel widely used in electric power transformers (Figure 1b). The coil couples to the magnetic field generated by the AC current flowing in one conductor of a conventional overhead line [5].

We made bench-test measurements of the power outputs of such coils when resistively loaded and placed them, adjacent their long sides, next to a stranded aluminum conductor like those typically used in 12,400-Volt overhead power-distribution lines. AC currents up to 100 A_rms_ (rms: root mean square) were used in our tests. In order to increase the harvester’s power output, we added electrical steel “flux guides” to the harvester.

Figure 1c,d show a very effective flux guide that covers the power-line conductor and the core of the coil, guiding to the coil’s core much of the magnetic flux that otherwise would not pass through the coil. (Note: the harvester, sensors and radio could be installed on an overhead powerline with a so-called “cherry-picker” or, as experienced power system technicians have suggested, for economy, one could do the installation with a telescoping insulated “hot stick”. When the harvester is being installed on a power line, the flux guide would be put over the conductor when the coil is nearly in place. It should be noted that an inexpensive means of installation, such as that described, is of considerable importance; in contrast, the installation cost for each of the large hard-wired sensor units in the 500-unit Chicago Array of Things project was about $3000 per sensor, according to a Project spokesperson.)

**Figure 2:**
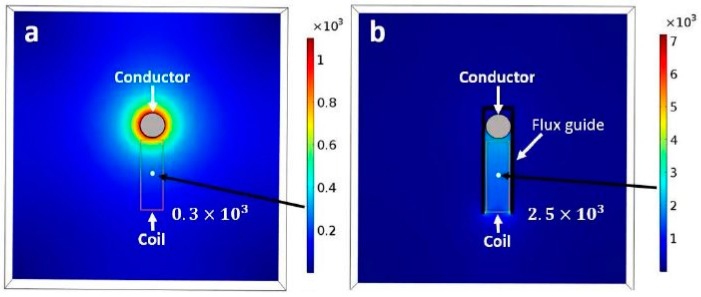
COMSOL simulation of the magnetic field intensity around the conductor without the flux-guide (**a**) and with the flux guide (**b**). The magnetic field intensity with the flux guide increases 8.33 times compared to no flux guide at the point illustrated by the light color dot (Note that the scales of the left and right figures are different).

The “flux guides” are sheets of strongly ferromagnetic material that guide magnetic fields from the space around the power-line conductor to the core of the coil. As shown in Figure 2, COMSOL modeling of the resultant 833% increase of the magnetic field intensity from the coil results from this flux guiding.

**Figure 3:**
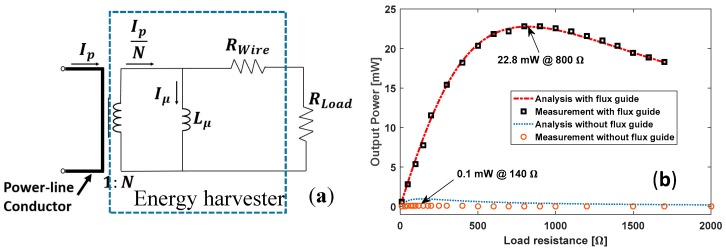
(**a**) Equivalent circuit of the energy harvester; (**b**) Measured output power vs. the load resistance for the energy harvester with and without using the flux guide. Parameters: *N* = 1250, $L_{\mu }$ = 2.15 H (with flux guide and 0.229 H without flux guide), $R_{wire}$ = 107 Ω.

Figure 3a shows an equivalent circuit of the energy harvester in the situation where the magnetic field in the core of coil does not saturate. The output terminals of the harvester are connected with a load resistor $R_{Load}$. The power delivered to the load resistance is given by

(1) $$P_{L}=\;\frac{{\left(I_{p}\omega L_{\mu }\right)}^{2}R_{Load}}{N^{2}\left[{\left(R_{wire}+R_{Load}\right)}^{2}+{\left(\omega L_{\mu }\right)}^{2}\right]}$$

where $L_{\mu }$, $R_{wire}$, and *N* are the inductance, resistance, and number of turns of the coil (and also turns of the secondary side of the current transformer), respectively. The frequency is set to 60 Hz, and $I_{p}$ is the rms current in the power-line conductor.

The maximum output power is

(2) $$P_{Lmax}=\;\frac{{\left(I_{p}\omega L_{\mu }\right)}^{2}}{2\;N^{2}\left[R_{Lopt}+R_{wire}/R_{Lopt}\right]}$$

at the optimal load resistance $R_{Lopt}$, given by

(3) $$R_{Lopt}=\;\sqrt{{R_{wire}}^{2}+\;{\left(\omega L_{\mu }\right)}^{2}}$$

Figure 3b shows the measured output power with and without using the flux guide as a function of the load resistance when the conductor current is 10 A_rms_. For the harvester without using the flux guide, the maximum output power of 0.1 mW is measured with the load resistor of 140 Ω. When the flux guide is used, the maximum output power of 22.8 mW is achieved when the load resistor is 800 Ω. By using the flux guide, the output power of the harvester increases more than *228 times* compared to the output with no flux guide.

**Figure 4:**
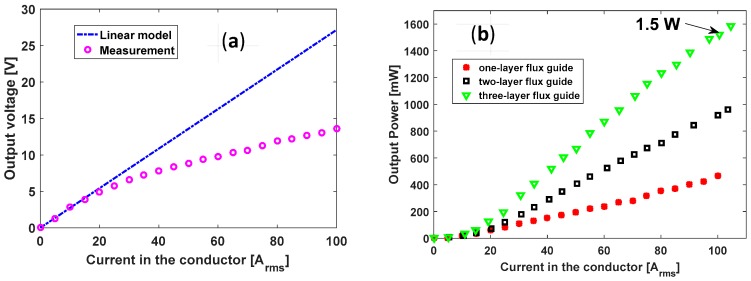
(**a**) Output voltage vs. current in the power-line conductor, $R_{L}$ = 400 Ω; (**b**) *Measured* output power vs. current in the power-line conductor, $R_{Load}$ = 400 Ω, 600 Ω and 1 kΩ for one-layer, two-layer and three-layer flux guides, respectively.

Figure 4a shows the output voltage vs. current in the simulated power-line conductor while the load resistance is fixed at 400 Ω. In the analytical model, the output voltage is proportional to the current in the conductor. When the current is larger than 20 A_rms_, the magnetic field in the coil starts saturating and the measured output voltage does not agree with the calculation from the analytical model. This is a result of the well-known phenomenon of magnetic saturation which occurs if the magnetic material is in a very large magnetic field, causing its magnetic property to be greatly reduced.

The output power of the harvester can be increased by using more layers of flux guide. The measured AC output powers of the harvester with one-, two- and three-layer flux guides are shown in Figure 4b. The measured AC power output was nearly *1.5 watts* when the simulated power-line current was 100 A_rms_, which is a fairly typical value for overhead power distribution lines [6]. The current on such a distribution power-line will vary with a varying load such as that due to the reduced power usage in dwellings. To avoid this, we have included an AC-to-DC (direct current) circuit, which contains a full rectifier and a capacitor, and an LTC 3130 (2.4 V–25 V input, 600 mA) Buck-Boost DC/DC Converter with the harvester, in order to ensure adequate system operation even though the power-line current changes. Note that one would also have to compensate for power intermittency when using intermittent power sources such as solar or wind.

**Figure 5:**
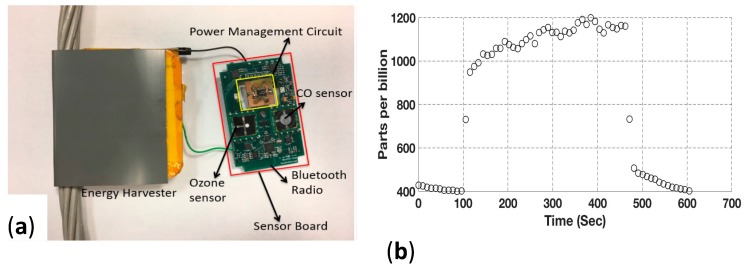
(**a**) Assembled harvester from Figure 1 sensor board showing ozone and carbon monoxide sensors and a Bluetooth radio antenna; (**b**) Measured ozone concentration from the sensor board.

The coil-based harvesters with this and other types of flux guides have produced powers sufficient for simultaneously operating a number of atmospheric sensors, as well as power-system sensors and radios to transmit data. As shown in Figure 5a, lab tests were made with a bare stranded power-line conductor, a harvester coil (yellow) with flux guide covering the conductor and the coil, a printed circuit board (PCB) containing ozone, carbon monoxide, temperature and humidity sensors, plus a Bluetooth radio. An LTC 3130 power management circuit (Linear Technology (Analog Devices), Milpitas, CA, USA) is lying on the PCB. Figure 5b shows the measured ozone concentration of 950 to 1200 parts per billion due to a 300-s indoor exposure from a Della commercial “air purifier” apparently designed for personal pulmonary relief. Data were transmitted to a nearby Android cellphone from the roughly 2 cm^2^ ozone sensor on the KWJ Engineering Sensor Board via the Board’s Bluetooth radio. During this test, the sensor board was powered from the harvester coupled to a conductor carrying a current of 17 A_rms_. The sensor board requires a maximum power of 16 mW to operate. In an outdoor test, the range of the Bluetooth radio was found to be 40 m.

## 3. Pollutants and Sensor Calibration

There are sources of public information about atmospheric properties but the data provided by many of those are qualitative (not quantitative, many are forecasts), not present values, and some combine atmospheric measures of several factors, such as particulate matter concentrations with ozone concentration to derive an air quality index, whereas some vulnerable people might prefer to learn the concentrations of one each pollutant independently, such as PM_2.5_, particulate matter whose aerodynamic diameter is 2.5 microns or less.

The spatial density of coverage of the existing atmospheric information sources is of interest. For example, the San Francisco Chronicle newspaper’s daily air quality *forecasts* contain 46 forecasts over an area of about 20,979 km^2^, for a density of only 0.0019 forecasts per square kilometer. As an example of the present density of high-quality pollution monitors, we note that there are 308 and 88 high-quality particulate matter PM_2.5_ and CO monitors, respectively, in California. This is equivalent to 0.00073 and 0.00021 PM_2.5_ and CO monitors, respectively, per square kilometer in California (area 423,970 km^2^). In contrast, with reasonable assumptions based on a nominal 61-m Bluetooth radio range, the density of the multi-sensor installations described in this study could reach 453 sensor installations per square kilometer (for a thorough discussion of the need for an increased density of atmospheric sensors see [7,8]).

The sensors powered by the energy harvesters will need to be tested and calibrated in calibration facilities when received from their vendors before and after their installation. Later, we expect to perform periodic calibrations, such as those for a particular pollutant, by comparing the responses of each installed sensor to those of a calibrated sensor similarly mounted on a hot stick held nearby, so that both sensors are exposed to the same pollutant concentrations and ambient atmospheres. In this way, we could derive a correction factor for each installed sensor that could be applied to that sensor’s reporting.

## 4. Atmospheric Sensors to Consider Deploying

According to the EPA (United States Environmental Protection Agency) [9], the Clean Air Act (CAA) requires EPA to set National Ambient Air Quality Standards (NAAQS) for six common air pollutants: carbon monoxide, ground-level ozone, lead, nitrogen oxides, particulate matter, and sulfur dioxide. Research in literature also shows the impacts of particulate matter and ozone to health [10,11].

We suggest that at least pollutants in the following list should be considered for future monitoring:

In addition to these pollutant sensors, one should include air temperature and humidity sensors in order to correct the responses of typical particulate matter and other sensors.

It is estimated that together the atmospheric sensors, listed in Table 1, can consume 203–303 mW. If instead of using the energy harvester as the power source, one uses a high-density battery, such as an ultraLife U9VL-J-P-10CP 9 V 1200 mAh Lithium (LiMnO_2_) battery to power sensors continuously, it would last only 36–53 h. In contrast, the coil-based energy harvester, which should output *1 watt* DC (assuming the efficiency of power conversion from AC to DC is 66%), can power the sensors for an indefinitely long time.

## 5. Power-System Sensors to Consider Deploying

In addition to atmospheric sensing, one could sense and report to power-system operators various operating variables of the power system itself. While solutions already exist for ways to sense some of the following power-system properties, the use of additional small sensors, powered by the energy harvester and using radios to report some of these properties, may be of interest to power-system operators.

Power-system properties whose measurement and reporting could be enabled, and for which sensors have been conceived, include the following: power-line current; power-line voltage (measured with a small sensor mounted on only one of the lines); instantaneous power flow (product of measured current and voltage); instantaneous direction of power flow on conductors in power systems that contain intermittent energy sources (solar, wind, or tidal); conductor temperature (important for determining on which circuits the power transmitted could safely be raised without increasing the sag of the line); acceleration to detect power-line structural collapse; conductor inclination and torsion [12]; and power-line intrusion by foliage.

## 6. Conclusions

We have described a small, coil-based energy harvester that couples magnetically to the time-varying alternating current flowing in a conductor of an overhead AC power distribution line to which it is attached. Lab testing has shown that an AC power of approximately *1.5 W* can be obtained when the harvester is placed near a power-line conductor carrying a 100 A_rms_ current. Such an energy harvester could power many different atmospheric sensors at once to determine the present local concentrations of numerous atmospheric pollutants, and the sensed concentrations could be broadcast to the cellphones of people nearby. Brief intermittent broadcasts with longer-range radios would permit the remote assembly of atmospheric pollutant maps for a large area. In addition, the harvester could supply energy to sensors that measure power-system quantities of interest to the system operators.

## Figures and Tables

**Table 1:** Selected pollutants, reasons for their inclusion, and estimated power drains required for their measurement. Note: Power consumption powers listed represent values for continuous operation; values for intermittent operation are obtained by multiplying the values listed by the fraction of time the sensors are functioning.

Pollutant	Continuous-Use Power (Estimated)
PM_2.5_^3^	200–300 mW
CO (carbon monoxide) ^3^	≤50 μW ^1^
Ozone ^3^	≤50 μW ^1^
NO_2_ (nitrous oxide) ^3^	≤50 μW ^1^
SO_2_ (sulfur dioxide) ^3^	≤50 μW ^1^
CO_2_ (carbon dioxide)	3 mW ^2^
Total estimated power consumed in continuous use	203–303 mW

^1^ SPEC Sensors. ^2^ GSS (Gas Sensing Solution) Sensors. ^3^ EPA (Criteria Air Pollutants).
